# Role of Spatially Fractionated Radiotherapy (LATTICE) Treatment in Inoperable Bulky Soft-Tissue Sarcomas

**DOI:** 10.3390/cancers17040624

**Published:** 2025-02-13

**Authors:** Katarina Majercakova, Natalia Tejedor Aguilar, Josep Isern Verdum, Helena Vivancos Bargalló, Antonio Vila Capel, Miriam Mancera Soto, Guillermo Gómez de Segura Melcón, Jady Vivian Rojas Cordero, José Antonio González-López, Silvia Bagué Rosell, Diana Hernandez Jover, Saba Rabi Mitre, Ana Peiró Ibañez, Ana Sebio, Gemma Sancho-Pardo

**Affiliations:** 1 Department of Radiation Oncology, Hospital de la Santa Creu i Sant Pau, Calle Sant Quintí 89, 08041 Barcelona, Spain; jisern@santpau.cat (J.I.V.); avilaca@santpau.cat (A.V.C.); jrojasc@santpau.cat (J.V.R.C.); rsaba@santpau.cat (S.R.M.); gsancho@santpau.cat (G.S.-P.); 2 Department of Radiophysics and Radioprotection, Hospital de la Santa Creu i Sant Pau, Calle Sant Quintí 89, 08041 Barcelona, Spain; ntejedor@santpau.cat (N.T.A.); hvivancos@santpau.cat (H.V.B.); mmancera@santpau.cat (M.M.S.); ggomezd@santpau.cat (G.G.d.S.M.); 3 Department of General and Digestive Surgery, Hospital de la Santa Creu i Sant Pau, Calle Sant Quintí 89, 08041 Barcelona, Spain; jgonzalezl@santpau.cat; 4 Department of Pathological Anatomy, Hospital de la Santa Creu i Sant Pau, Calle Sant Quintí 89, 08041 Barcelona, Spain; sbaguer@santpau.cat; 5 Department of Radiology, Hospital de la Santa Creu i Sant Pau, Calle Sant Quintí 89, 08041 Barcelona, Spain; dhernandez@santpau.cat; 6 Department of Orthopaedic Surgery and Traumatology, Hospital de la Santa Creu i Sant Pau, Calle Sant Quintí 89, 08041 Barcelona, Spain; apeiro@santpau.cat; 7 Department of Medical Oncology, Hospital de la Santa Creu i Sant Pau, Calle Sant Quintí 89, 08041 Barcelona, Spain; asebio@santpau.cat

**Keywords:** sarcoma, SFRT, LATTICE LRT, GRID

## Abstract

**Background**: The clinical outcome of inoperable sarcoma patients treated with LATTICE (LRT) is limited and therefore the objective of our study was to report treatment response, overall survival (OS), local-recurrence free survival (LRFS) and toxicity. **Methods**: This retrospective observational study includes 15 histologically proven inoperable non-extremity sarcoma patients with no treatment options or no response to systemic therapy, treated at our institution between 2020 and 2024. The patients were treated with a combination of LRT and normo- or hypo-fractionated external beam radiotherapy. Treatment response was evaluated by RECIST1.1 criteria, toxicity by CTCAE 5.0 and OS and LRFS by Kaplan–Meier curves. **Results**: The median follow-up (F-UP) since the beginning of the treatment was 10 months (range 4–32). Nine patients were male and six female. Their mean age was 60 years. The median gross tumor volume (GTV) was 1058 cm^3^ (range 142–6103 cm^3^). The median number of spheres was 9 (4–30). All patients with symptoms reported symptoms’ relief. Based on RECIST1.1 criteria, 10 patients (67%) had stable local disease at 1–2 months F-UP on computed tomography (CT). Surgical resection was feasible in five patients. Three of them are alive without disease and two died due to metastatic progression. From 10 (67%) non operated patients, 5 patients died (50%) due to disease. The remaining five patients (50%) are alive, three with stable disease at 21, 22, and 32 months of F-UP and two with disease progression who are currently receiving palliative chemotherapy treatment. Reported G2 toxicity was as follows: gastrointestinal (2), asthenia (1). Two patients had G3 toxicity: esophagitis (1) and inguinal dermatitis (1). No acute or chronic G4–G5 toxicity was observed. **Conclusions**: LRT is a feasible and well-tolerated radiation technique for inoperable bulky soft-tissue sarcomas. Further studies are needed to establish protocols to determine which patients could benefit from palliative or preoperative treatment.

## Simple Summary

Surgery is the standard care treatment of sarcomas, but the clinical outcome of inoperable bulky tumors is poor. Therefore, due to tumor characteristics, spatially fractionated radiotherapy (SFRT) might be a treatment option in these cases. LATTICE (LRT) is a technique of SFRT that allows a high dose of radiation (10–20 Gy) to be administered at small volumes inside a tumor, while simultaneously reducing the dose to normal tissue. Currently, no treatment protocols are available and only a few studies have included sarcoma patients. Therefore, the main objective of our study was to report the clinical outcome of patients with inoperable, non-extremity sarcomas with no other treatment option treated with LRT.

## 1. Introduction

Sarcomas are rare tumors that represent ≤1% of all adult solid malignancies [1] and surgery plays the major role in their definitive treatment [2]. In inoperable cases, radiotherapy is an available alternative, but the treatment outcome for large tumors is poor [2].

Large tumor size and the presence of more hypoxic areas within the tumor require the administration of a higher dose in order to improve the treatment outcome [3]. However, delivering ablative doses using standard techniques is not feasible due to the high risk of toxicity to surrounding organs at risk (OARs). With the technical improvement in last decades, stereotactic body radiotherapy (SBRT) permits the administration of high doses to tumors, resulting in excellent treatment outcomes for relatively small lesions [4]. However, its implementation in tumors larger than 5 cm is discouraged due to higher risk of toxicity [5]. As a result, recently a “new–old technique’’ called spatially fractionated radiation therapy (SFRT) has been implemented for the treatment of bulky tumors larger than 6–10 cm [6,7].

Originally, in 1909, Kohler started to implement SFRT as 2D GRID therapy to decrease the skin dose in the treatment of deep tumors [8]. SFRT allows inhomogeneous high doses of radiation to be administered within the gross tumor, creating areas of high and low doses known as peaks and valleys. This approach has led to its implementation in the treatment of bulky tumors. GRID therapy can produce dramatic relief of severe symptoms [9]. Recently, preclinical studies have reported that these ablative doses have an impact on the immune system, leading to effects such as the bystander effect, the abscopal effect and microvascular changes [10]. These effects are being further investigated. With the implementation of advanced techniques, such as intensity-modulated radiotherapy (IMRT) and volumetric-modulated radiotherapy (VMAT), a 3D configuration of SFRT known as LATTICE (LRT) has started to be applied in clinical practice. This combination of modulated techniques and SFRT permits better preservation of OARs with limited toxicity [11,12] and a reduction in the dose to the skin [13].

Several recent studies suggest that SFRT treatment for primary tumors and metastatic lesions appears to be effective and safe [9,11,13]. However, most of these studies are case reports, and no protocols or guidelines have been established yet. Additionally, most of these studies have had limited representation of sarcoma patients with non-extremity localizations [12]. Therefore, the main objective of this study is to evaluate the acute toxicity, the symptoms’ relief and treatment outcome in patients diagnosed with inoperable non-extremity sarcomas with no option of other active treatment, including patients with failure to previous treatments.

## 2. Materials and Methods

This retrospective observational study includes 15 patients who had no other treatment options or did not respond to systemic therapy, treated at our institution between 2020 and 2024 with palliative intention. All cases were evaluated by the tumor board prior to the treatment.

### 2.1. Treatment Planification

Treatment planification was performed on computed tomography (CT) with 2–3 mm axial slice thickness. Intravenous (i.v.) and oral contrast were administered when necessary. After delineation of internal target volume (ITV) or gross-tumor volume (GTV), a 5 mm axial margin was added to create the clinical target volume (CTV), while excluding OARs. Additionally, a 5–7 mm margin was added to establish the planning target volume (PTV)). Patients with good performance status (PS 0–1) received normofractionated external beam radiotherapy (EBRT) with a prescription dose of 45–54 Gy (EQD_2_) and those with PS 2 were treated with short course treatment of 20–25 Gy/4–5 Gy or 30 Gy/3 Gy per fraction.

Treatment planning was carried out by experienced radiotherapists and physicists, following a protocol based on the guidelines of Grams et al. [14]. PET-TC was not routinely performed. LRT treatment was administered before or after EBRT in a single fraction of 20 Gy to 1.5 cm diameter spheres placed within the ITV/GTV outlined on the planning CT scan, ensuring a separation of at least 3 cm from center to center and at least 1 cm from OARs. Three concentric volumetric rings around the spheres, with margins of 0.5 cm, 1 cm and 3 cm were generated during the optimization process. These rings had different maximum dose objectives of 98%, 50% and 40%, respectively, in the optimizer to concentrate the highest doses at the center of the spheres while maximizing dose sparing between them. In general, it is not necessary to include the organs at risk (OARs) in the optimizer, as they receive low doses due to their distance from the spheres. Additionally, the high dose gradient created by the concentric rings effectively minimizes exposure outside the GTV/ITV.

Treatment plans were optimized and calculated using the Acuros XB (dose to medium) algorithm with the Eclipse v.15.6 TPS (Varian Medical Systems, Palo Alto, CA, USA), employing the VMAT technique with 6 MVFFF energy arcs and a dose rate of 1400 MU/min. In one patient, 10 MVFFF energy and a dose rate of 2400 MU/min were employed due to its larger size and to shorten treatment time.

The optimization was considered complete when the following dosimetric criteria are met: the D_50%_ of each sphere is within ±50 cGy of the prescription dose (PD), a maximum dose in the spheres below to 140% of the PD and the dose bridges between spheres are approximately 30–40% of the PD. OARs constraints for the combined plans (LRT and EBRT) were discussed and evaluated in EQD_2_ by both physicist and radiation oncologist.

The pre-treatment verification was performed using the electronic portal imaging device (EPID) and evaluated with Varian Portal Dosimetry. The metric used for analysis was a global gamma criteria (3%, 2 mm) with a threshold of 10% and a passing rate above 90%. Additionally, for this type of treatment, a pre-treatment verification was performed using a Quasar Respiratory Motion phantom (Modus Medical Devices) without the respiratory motion assembly. A Semiflex 0.125 cm^3^ ionization chamber (PTW) was positioned in a high-dose region (center of a sphere) and a low-dose region (between spheres). The relative dose difference between the calculated plan and the measurements should be less than 5%.

**Figure 1:**
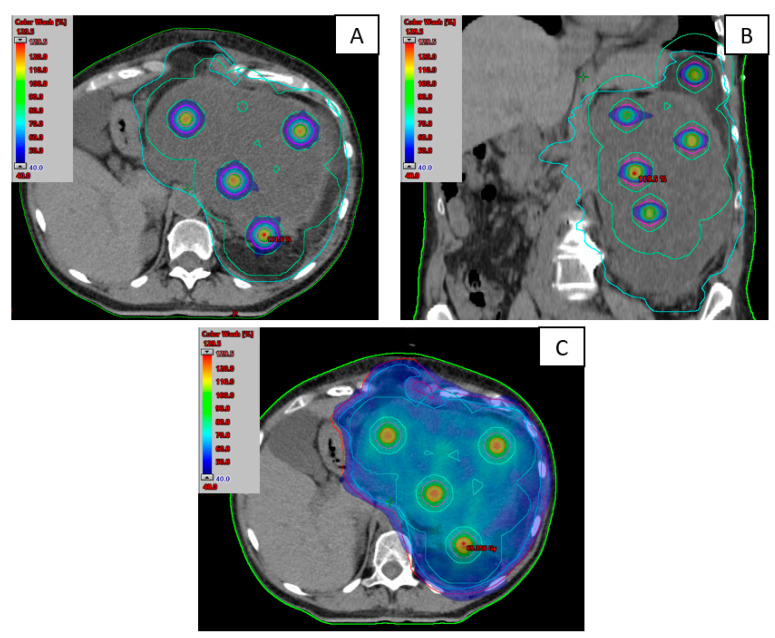
Dose distributions of LATTICE (LRT) planification in axial (**A**) and coronal view (**B**). Dose distribution of the total plan (EBRT + LRT) in an axial view (**C**). The following structures are shown: CTV (light blue), spheres (red) and the concentric rings for optimization (light green and magenta).

Treatments were delivered in a Truebeam sTx linear accelerator with Millenium 120 MLC (Varian Medical Systems, Palo Alto, CA, USA) and equipped with the AlignRT system (Vision RT). See an example of treatment planification in Figure 1.

### 2.2. Clinical Evaluation

Daily cone beam CT (CBCT) was performed to ensure patient positioning. On the day of the LRT, the CBCT was reviewed by the radiation oncologist to ensure that the target regions are within the ITV/GTV and sufficiently distant from the OARs. Additionally, surface-guided radiotherapy (SGRT) using AlignRT version 6.3 was performed to monitor patient intrafraction motion. When anatomical changes such as weight loss, tumor shrinkage or (pseudo)progression were observed, an offline adaptive approach was implemented.

Clinical response was evaluated by RECIST1.1 criteria. Toxicity was evaluated using the CTCAE 5.0 scale. Symptom relief was assessed on a scale of relief, no relief or worsening of the symptoms. The first evaluation of treatment response was performed by CT between 1 or 2 months after the end of the treatment and if no progression was observed every 3 months afterwards. Local progression free survival was defined as the time from the beginning of the treatment until the event occurred and was evaluated using Kaplan–Meier curves.

## 3. Results

### 3.1. Patients’ Characteristics

A total of 15 patients, 6 female and 9 males, were included in the study. The median age was 60 years (range, 29–81). The median tumor size was 13.5 cm (range 8–40). The median follow-up (F-UP) was 10 months (range, 4–32). The majority of the treated lesions were primary tumors (73%). A total of 80% of lesions were localized in the abdominopelvic area, while 20% were in the thorax. Most of them were rapidly progressive tumors. One patient with a chest wall–thorax lesion was previously irradiated with hypofractionated radiotherapy in the same area. The most common histologies were undifferentiated spindle cell sarcoma and dedifferentiated liposarcoma (47%). No concomitant chemotherapy or immunotherapy were administered. The median GTV/ITV volume was 1058 cm^3^ (range, 142–6103). See the patients’ characteristics in Table 1.

### 3.2. Treatment Characteristics

The median number of spheres was 9 (range 4–30). Fourteen patients received 20 Gy in a single fraction with LRT. Only one patient with a bulky tumor lesion in the thoracic area and significant dyspnea received five fractions of 13.5 Gy. Regarding EBRT, nine (60%) patients with PS 1 received 45–54 Gy (2 Gy per fraction), five (33%) patients with PS 2 received a hypofractionated dose of 20–25 Gy (4–5 Gy per fraction) and one (7%) patient with PS 2 and bulky paraesophageal lesion received 30 Gy (3 Gy per fraction).

The median time between LRT and EBRT treatment was 1 day. Nine patients started EBRT one or two days after LRT, while five patients started between five and nine days later. Only 1 patient received the EBRT 14 days after EBRT because initially an IGABT boost was indicated, but was later switched to LRT treatment. Table 2 shows treatment parameters for all patients, and Figure 1 illustrates an example of one patient’s dosimetric plan for LRT (a and b) and the total treatment plan (c).

All patients were treated with the VMAT technique. Thirteen (87%) patients received LRT treatment followed by EBRT. Only two (13%) patients were treated with a different regimen: The first one received LRT as a boost technique after EBRT. The patient’s tumor was in the pelvic cavity and initially image-guided brachytherapy (IGABT) was proposed; it was not technically possible due to tumor depth and changing the LRT treatment was proposed. The second patient had multiple symptoms such as hypoalbuminemia and anaemia, which required frequent blood transfusion. Given the urgency of the situation, EBRT was started while the LRT treatment was prepared. Therefore, the SFRT was administered during the EBRT treatment.

Replanning was performed in two patients: the first due to weight loss and tumor reduction, and the second because two lesions were treated with LRT during the same treatment course. The first lesion was treated before beginning the normofractionated regimen, while the second lesion, located near the heart, was treated during the EBRT course due to progression observed on the CBCT.

### 3.3. Treatment Outcome

Eleven (73%) patients had symptoms depending on tumor localization such as pain, constipation, dyspnea, edema, anemia and asthenia at the beginning of the treatment. All of them reported improvement in symptoms during the treatment. Based on RECIST1.1 criteria, 10 (67%) patients had stable disease, while 5 patients (33%) local progression at first F-UP at 1–2 months. See Table 3.

**Figure 2:**
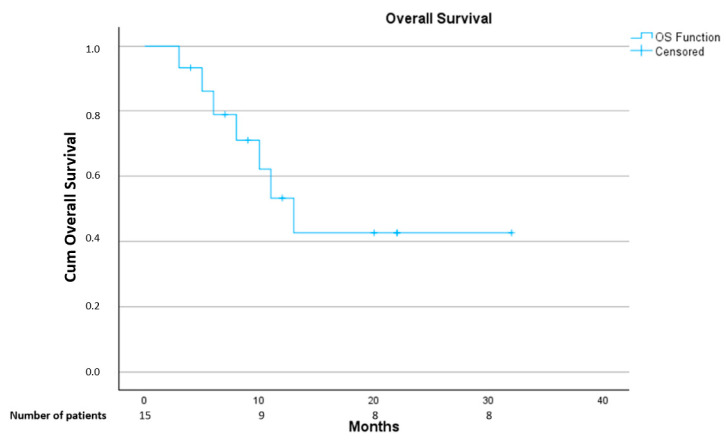
Overall survival. Kaplan–Meier curve.

**Figure 3:**
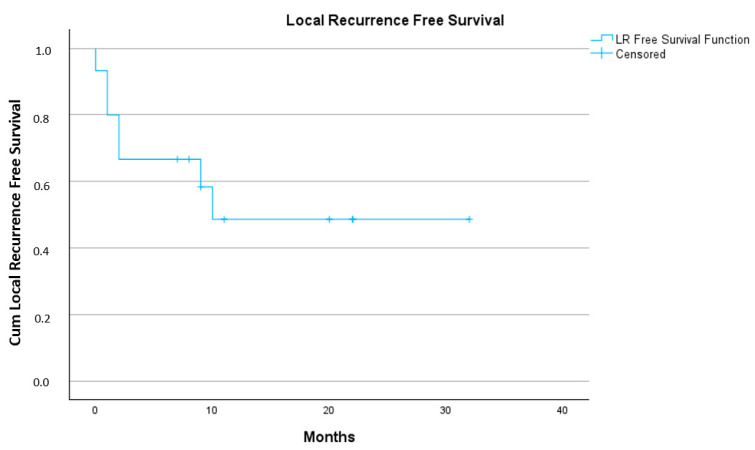
Local recurrence free survival. Kaplan–Meier curve.

One-year OS rate was 43% and one-year local recurrence free survival rate was 49%. See Figure 2 and Figure 3.

Of the five (33%) patients with symptoms’ relief and stable disease by RECIST1.1 criteria who after the tumor growth stabilization could be operated on, two died: one due to pulmonary progression 11 months after RT and the other due to peritoneal and local progression (tumor rupture during the surgery was reported) 13 months after the RT. The remaining three operated patients are alive and disease-free at 7, 9 and 20 months F-UP. One of them, with multiple symptoms, including anemia that required various blood transfusions, before beginning the treatment, urgent EBRT had been started, LRT was administered during the treatment and replanification was required due to tumor reduction and weight loss. The patient’s symptoms disappeared with a 27% tumor reduction on CT. Subsequently, the patient underwent surgery; however, only a 10% pathological response was reported. The patient is disease-free at the last F-UP (20 months).

From ten (67%) non operated patients, five patients died (50%): one due to local progression, three due to both local and metastatic progression and one due to intestinal occlusion because of extrinsic tumor compression. The remaining five patients (50%) are alive: three with stable disease at 21, 22 and 32 months of F-UP and two with disease progression, who are currently receiving palliative chemotherapy treatment.

No differences in clinical or radiological responses were detected according to RECIST 1.1 criteria between the different doses. All patients demonstrated a clinical response, regardless of the EBRT dose received. Among the five patients with local progression at the first follow-up, three had received 45–54 Gy in 25–27 fractions and two had received 20 Gy in five fractions. However, all operated patients with stable disease, as per RECIST 1.1 criteria, had received 45 Gy. Of the three long-term survivors with stable disease, two received 50 Gy, while the remaining patient received 25 Gy in 5 fractions.

### 3.4. Toxicity

No ≥G4 toxicity was reported. Five (33%) patients had no toxicity, and ten (67%) patients had at least one G1–G2 toxicity. The most reported toxicity was G1–G2 gastrointestinal (GIT) toxicity such as diarrhea, nausea or bloating, affecting 40% of patients. Only two patients had G3 toxicity: One of the patients, who had a bulky paraesophageal lesion along the entire esophagus, was treated with 30 Gy in 10 fractions after LRT therapy. This patient developed G3 esophagitis, which required hospitalization and parenteral nutrition. No ulceration on gastroscopy was observed and nasogastric tube was removed. Posteriorly, the patient experienced single lung metastasis treated with SBRT, and renal metastasis treated with cryoablation. The patient is currently receiving chemotherapy treatment due to local progression 8 months after LRT. The second patient with inguinal-pelvic chondrosarcoma reported G3 inguinal skin toxicity with local and tumor infection that required drainage and intravenous antibiotic treatment. The patient’s tumor progressed 2 months after the end of treatment. See more details in Table 3.

## 4. Discussion

LRT can be administered as a unique treatment in 1–5 fractions [7,15,16] or in combination with normo-or hypofractionated EBRT, either as consecutive treatments or as an integrated boost [13,17,18,19]. However, standardized protocols or recommendations are not available yet for treatment planning or patients’ selection. Recently, a consensus was published on the design of prospective clinical−translational trials in SFRT in head and neck and sarcoma cancer patients, but non-extremity sarcomas and more radioresistant sarcomas such osteo- or chondrosarcomas were not included [20].

The majority of published studies are case reports or retrospective studies with lung and gynecological tumors being the most frequently treated [9,12]. The representation of sarcomas in these studies is poor, as they are rare and highly heterogeneous tumors. [13,18,21]. Additionally, they are considered as radioresistant tumors, requiring a higher dose to be treated. However, some studies suggest that, due to sarcoma characteristics, patients might benefit from SFRT (LRT/GRID) treatment [22]. In a recently published study, the spheres have been placed in necrotic areas with lower SUV activity in PET-TC and clinical results are promising [18].

The primary objective of the SFRT studies published to date has been symptom relief, which was achieved in all of our patients during the follow-up period. The results are consistent with the available literature [12,18,23]. Some studies have also reported an important tumor reduction, but most of them included non-sarcoma histologies that are more sensible to radiotherapy [12]. Recently, initial studies evaluating SFRT (GRID or LRT) in sarcoma patients have reported tumor responses ranging from 76 to 91% [23,24,25]. The series in these studies are heterogenous, with some focusing only on extremity sarcomas [24], and others including various tumor sites [23,25]. Additionally, some patients also received concomitant chemotherapy [24]. Furthermore, the criteria for evaluating response are not uniform, with some studies classifying responses as complete, partial (any reduction in tumor size) or tumor progression [23]. Our treatment response was evaluated based on RECIST1.1 criteria at 1–2 months after the end of treatment. Only one patient had a 27% tumor reduction, categorized as stable disease by RECIST1.1 criteria. However, five patients classified as having a stable disease by RECIST1.1 criteria underwent surgery and showed 10–90% pathological tumor necrosis. Until now, only few studies or case reports where SFRT treatment was administered preoperatively are available, reporting a pathological response ranging from 0 to 100% [24,26,27,28]. To the best of our knowledge, this is the first study where 33% of abdominopelvic sarcoma patients turned to be operable after LRT, suggesting that some selected patients might benefit from neoadjuvant SFRT. We have observed discrepancies between clinical, radiological and pathological responses, indicating that other evaluation criteria, for example Choi criteria, including size and tumor density on CT, should be considered in assessing treatment response [29].

Additionally, the timing for clinical evaluation remains unclear. Published studies show variability in the timing of the first radiological assessment; however, recent consensus recommends evaluation within 1–2 months [20]. In our study, patients were evaluated by CT at 1–2 moths F-UP. It should be noted that three of our non-operated patients with stable disease and no chemotherapy administration, who are long-term survivors, showed maximum treatment response at 2, 7 and 19 months of F-UP. Therefore, a consecutive evaluation is crucial.

In recent published studies including only sarcoma patients treated with SFRT, the 1-y LC and 1-y OS rates were 82% and 53% respectively [23], whereas in our study were 52% and 42%, respectively. One of the reasons could be that the median tumor volume in our cohort was significatively larger (1058 cm^3^) in comparison to reported studies with 146.48 cm^3^ by Ferini et al. [18] or 636 cm^3^ in Ahmed et al. [23]. Further, no concomitant chemotherapy or immunotherapy was administered in our study. Tumor location may also have an impact on treatment outcomes, as 80% of our patients had abdomen–pelvic tumors in comparison to 52% in the study by Ahmed et al. [23]. Another difference is the treatment planning method, which was conducted following a conservative approach [14], but no G4–5 toxicity was observed in comparison to other studies that reported tumor lysis, fistulae or perforation [11,23].

Overall, a single dose of LRT at 15–20 Gy and normofractionated EBRT seems to be safe, with most studies reporting only 20% of G1–G2 toxicities [23]. Our study exclusively includes non-extremity sarcoma patients with different histologies treated with LRT technique at a 20 Gy dose in spheres combined with either a normofraccionated or hypofractionated EBRT. The reported G1–2 toxicity in our study was 67%, with only two patients experiencing G3 toxicity. The first of these patients was diagnosed with a groin–pelvis chondrosarcoma and the EBRT dose was escalated to 50.4 Gy, as previous studies had shown that the administration of >50 Gy [25] led to improved treatment outcome. Skin tolerance in the groin area is generally poorer in comparison to other skin locations, which likely contributed to the toxicity, as reported in other previous studies [23,25]. The second patient with G3 toxicity had a paraesophageal mediastinal lesion and received 30 Gy in 10 fractions of EBRT. The lesion extended around the entire esophagus, making it difficult to determine whether the toxicity was due to LRT or EBRT. Additionally, in our LRT planification, OARs, including main vessels, nerves, bones and skin were carefully avoided, resulting in minimal dose exposure. Factors such as 40% of patients with PS 2, the predominance of lesions in abdominopelvic location, larger tumor volumes and hypofractionation may have contributed to the increased toxicity observed. Therefore, hypofractionated EBRT after LRT in the abdominopelvic area in patients with worse PS should be approached with caution.

Our study has several limitations, the main one being its retrospective nature. Regarding treatment planning, in the case of large tumor volumes, placing the spheres according to the guidelines of Grams et al. [14] made optimization impossible due to the high number of high dose regions. Therefore, in these cases, the number of spheres was reduced by increasing the spacing between them within the tumor. Additionally, it is important to note that our patient cohort is small, consisting of heterogenous sarcoma subhistologies, with the majority having lesions in the abdominopelvic region. Despite this, we have reported the largest number of operated abdominopelvic sarcoma patients previously treated with LRT published to date. Further, in our study QoL evaluation was not reported and the toxicity might be underreported due to reliance on physician reported outcomes [30].

## 5. Conclusions

SFRT treatment is generally well tolerated and the published results are promising. However, the treatment indication (palliative or preoperative), patient selection criteria, standardized treatment protocols and timing for treatment evaluation remain unclear. Further prospective studies are required to address these aspects, as some selected patients might benefit from planned preoperative SFRT.

## Figures and Tables

**Table 1:** Patients’ characteristics.

Characteristics (*n* = 15)	
Median age	60 (range 29–81)
Patient gender	Male: 9 (60%) Female: 6 (40%)
Number of lesions treated with LRT	1 lesion: 14 (93%) 2 lesions: 1 (7%)
Tumor type	Primary: 11 (73%) Metastasis: 4 (27%)
Median GTV/ITV volume (cm^3^)	1058 (range 142–6103)
Treatment site	Thorax: 3 (20%) Abdomen: 7 (47%) Pelvis: 5 (33%)
Histology	Chondrosarcoma: 2 (13%) Dedifferentiated liposarcoma: 4 (27%)
	Endometrial stromal sarcoma: 1 (7%) Leiomyosarcoma: 2 (13%) Malignant peripheral nerves heath tumor: 1 (7%) Synovial sarcoma: 1 (7%) Undifferentiated pleomorphic sarcoma: 1 (7%) Undifferentiated spindle cell sarcoma: 3 (20%)
Performance status (PS)	0–1: 9 (60%) 2: 6 (40%)

**Table 2:** Treatment parameters.

Treatment Parameters	Median (Range)
Number of spheres	9 (4–30)
LRT dose (Gy)	20
EBRT dose (Gy)	45 (20–54)
Number of EBRT fractions	25 (5–27)
Number of days between LRT and EBRT	1 (1–14)

**Table 3:** Clinical results and toxicity.

Clinical Results and Toxicity	
Number of patients with symptoms before treatment	11 (73%)
Clinical response	No: 0 Yes: 11 (100%) Worsening: 0
Number of patients operated	5 (33%)
RECIST1.1 criteria	Stable disease: 10 (67%) Progression: 5 (33%)
Toxicity G0	5 (33%)
Toxicity G1	Skin: 1 (7%) Gastrointestinal: 4 (27%) Genitourinary: 2 (13%)
Toxicity G2	Gastrointestinal: 2 (13%) Asthenia: 1 (7%)
Toxicity G3	Gastrointestinal: esophagitis: 1 (7%) Skin: 1 (7%)
Pathological response of operated patients (median, range)	45% (10–90%)

## Data Availability

Data are contained within the article.
